# Commentary: Methamphetamine mediates immune dysregulation in a murine model of chronic viral infection

**DOI:** 10.3389/fmicb.2015.01473

**Published:** 2015-12-23

**Authors:** Jennifer M. Loftis

**Affiliations:** ^1^Veterans Affairs Portland Health Care System, Research and Development ServicePortland, OR, USA; ^2^Department of Psychiatry, Oregon Health and Science UniversityPortland, OR, USA; ^3^Methamphetamine Abuse Research Center, Oregon Health and Science UniversityPortland, OR, USA

**Keywords:** addiction, alcohol, blood brain barrier, co-morbidity, chronic viral infection, hepatitis C, methamphetamine, T cells

The recent article by Sriram et al. (2015) is one of the first studies to evaluate the effects of methamphetamine on peripheral T cell responses, in the context of a chronic viral infection. Using lymphocytic choriomeningitis virus (LCMV) clone 13 as a model of chronic viral infection in mice, investigators found that exposure to methamphetamine significantly alters the expression of peripheral T cell factors putatively involved in infection control. In particular, the expression of programmed cell death protein-1 (PD-1), type 3 inflammatory CXC chemokine receptor (CXCR3), and epidermal growth factor receptor (EGFR) was increased on T cell subsets from LCMV-infected mice exposed to methamphetamine. Collectively, these immune cell changes have the potential to adversely impact key anti-viral immune functions, such as T cell recruitment, cytokine production, and exhaustion.

Given the high prevalence of chronic viral infections [e.g., human immunodeficiency virus (HIV) and hepatitis C virus (HCV)] associated with methamphetamine and other substance use disorders, including alcohol (Martínez-Raga et al., 2001), it is important to determine how substances of abuse effect T cell responses involved in the etiology and progression of chronic viral infection. Co-morbid substance use disorders and HIV and/or HCV infections are also often associated with impairments in central nervous system (CNS) function (Carey et al., 2006; Loftis et al., 2006; Butt et al., 2007; Fuller et al., 2009), which can hinder successful treatment outcomes (Fals-Stewart, 1993; Aharonovich et al., 2003). Similarly, infectious symptoms (e.g., fatigue, pain, and depression), potentially caused by substance abuse-related peripheral immunosuppression, could lead to further likelihood of relapse or increased substance use. Indeed, the tendency of adults with substance use disorders to self-medicate with alcohol and other drugs of abuse (Bolton et al., 2009; Crum et al., 2013) could be recognized as a possible complication of untreated or uncontrolled viral infection symptoms. These and other findings highlight the converging effects of substances of abuse on immune function—effects that contribute to the addiction and increase susceptibility for the progression of chronic viral infections. Despite changes in peripheral T cell markers associated with viral clearance, Sriram et al. (2015) reported that methamphetamine exposure did not significantly alter viral loads in plasma or tissues (i.e., spleen, lungs) from mice with LCMV clone 13 infection. However, it would be of interest to investigate whether or not methamphetamine exposure contributed to the invasion of virus into the brain, as LCMV (although considered to be peripherally restricted) can infect the brain and cause CNS impairments, including learning deficits (Brot et al., 1997).

Methamphetamine and other substances of abuse alter blood brain barrier (BBB) function (Haorah et al., 2008; Kousik et al., 2012; Northrop and Yamamoto, 2012) and potentially increase the invasion of peripheral viruses, such as HIV and HCV, into the brain (e.g., Gavrilin et al., 2002; Bokemeyer et al., 2011). In a previous study using a murine model of HIV-1 encephalitis, Potula et al. (2006) innovatively demonstrated the presence of CD8+ T cell infiltration in brain areas with HIV-1 monocyte-derived macrophage infection and importantly, the impairing effects of alcohol on viral clearance. Emerging evidence suggests that T cells may play a critical role in these processes and in the development of CNS damage resulting from viral infection and co-morbid substance abuse (Gaskill et al., 2013; Coley et al., 2015). It is noteworthy that Sriram et al. (2015) observed increased expression of T cell markers, which among other immune functions, play a role in the transmigration of T cells into the CNS. For example, studies show a role for CXCR3 in CD8+ T cell trafficking in the brain following intracranial LCMV infection in mice (e.g., Christensen et al., 2006), and CXCR3 has been proposed to serve as a surface marker for cells that have the ability to cross the BBB (Callahan et al., 2004). Similarly, in addition to regulating the sensitivity to alcohol (Corl et al., 2009), EGFR activation contributes to the integrity of tight junctions between brain endothelial cells (Chen et al., 2011), and tight junctions are one of the most important structural elements of the BBB. Thus, substance use disorders may exacerbate the increased trafficking of peripheral monocytes to CNS, and in combination with compromised BBB function, may have significant consequences for individuals with co-morbid viral infection(s) (e.g., HCV and/or HIV).

As an initial step to identify mechanisms by which chronic viral infection and alcohol (ethanol; EtOH) induce abnormalities in T cell function, potentially facilitate neuroinvasion of LCMV, and contribute to CNS impairments, we exposed BALB/c mice to EtOH and water (or water only) using a two-bottle choice paradigm, followed one week later by infection with either LCMV clone 13, LCMV Armstrong (causes acute infection), or vehicle. Mice were monitored for 60 days post-infection and continued to receive 24-h access to EtOH and water. Consumption of EtOH was associated with alterations in virus-specific CD8+ T cell expression and delayed viral clearance in mice with LCMV clone 13 infection (Loftis et al., 2015). Research to determine the effects of LCMV and alcohol exposure on CNS viral invasion and behavioral outcomes is in progress. Sriram et al. (2015) aptly note that; “LCMV has proven to be a great model to study chronic infections in mice as they induce a robust T cell response” (p. 8). Given that persistent CNS infection can lead to the generation of autoimmune responses and that the presence of viral proteins in the CNS can increase sensitivity to and susceptibility for substance abuse (Vigorito et al., 2015), more research is needed to provide a better understanding for the molecular basis of the neurotoxic combination of substance abuse and chronic viral infections, such as HCV and HIV. These investigative efforts will be instrumental in translating basic science and preclinical findings into clinical practice. For example, development of a database of infectious complications associated with substance use disorders could be established. Through the use of such a database (e.g., listing co-morbid HCV infection and opioid use disorder complicated by acute bacterial endocarditis, Wakeman et al., 2014), we may be better able to correlate key clinical observations with research findings, leading to more targeted (and potentially more aggressive) infection treatment strategies for patients with co-morbid substance use disorders.

## Funding

This material is the result of work supported with resources and the use of facilities at the Veterans Affairs Portland Health Care System, Oregon Health & Science University, and the Methamphetamine Abuse Research Center (NIDA P50DA018165), Portland, Oregon, USA. This work was supported by the U.S. Department of Veterans Affairs Biomedical Laboratory Research and Development Merit Review grant (#I01 BX002061) to JL. The contents do not represent the views of the U.S. Department of Veterans Affairs or the United States Government.

### Conflict of interest statement

The author declares that the research was conducted in the absence of any commercial or financial relationships that could be construed as a potential conflict of interest.
